# Comparison of avascular lymph node fragment transplantation techniques to optimize lymphangiogenesis in the minipig model

**DOI:** 10.1007/s00238-021-01869-3

**Published:** 2021-10-12

**Authors:** Catarina Hadamitzky, Frank Bruns, Klaus-Friedrich Gratz, Lia Schindewolffs, Katrin S. Roth, Martin Werner, Kristiana Gordon, Peter M. Vogt, Reinhard Pabst

**Affiliations:** 1 Department of Plastic, Aesthetic, Hand and Reconstructive Surgery, Hannover Medical School, Hannover, Germany; 2 Hannover, Germany; 3 Department of Radiation Oncology, Hannover Medical School, Hannover, Germany; 4 Department of Nuclear Medicine, Hannover Medical School, Hannover, Germany; 5 Institute of Functional and Applied Anatomy, Hannover Medical School, Hannover, Germany; 6 ZRN Rheinland, Center for Radiology and Nuclear Medicine, Korschenbroich, Germany; 7 Lymphoedema Department, St. George’s Hospital, London, UK; 8 Institute of Immunomorphology, Hannover Medical School, Hannover, Germany

**Keywords:** Lymphangiogenesis, Lymphoedema, Vascular endothelial growth factor-C, Lymph node transplantation, Surgical technique, Porcine animal model

## Abstract

**Background:**

Secondary lymphoedema is a challenging pandemic. This condition may arise after oncologic resection of tumor-draining lymph nodes and/or radiation. Plastic-surgical procedures for lymphoedema comprise transplantation of vascularized lymph node flaps, which are, however, technically challenging and difficult to implement on a global level due to the scarcity of microsurgery facilities in some countries. To improve this situation, comparative research in valid animal models is needed.

**Methods:**

A total of 33 minipigs were subjected to lymphatic resection in the hind limbs. This large animal model was used in a first phase to compare different lymph node fragmentation methods and assess lymphatic regeneration after avascular transplantation. In a second phase, several stimulants were tested for their effect on lymphatic regeneration after fragment transplantation. In a third phase, animals additionally received irradiation of the groin. In this novel animal model, autologous avascular lymph node fragment transplantation was complemented by peripheral injections of vascular endothelial growth factor-C (VEGF-C). Finally, regeneration rates were quantified in relative numbers (percentage) in the irradiated tissue.

**Results:**

In the first phase, transversal lymph node fragmentation under preservation of the nodal capsule showed the best percentage of regeneration (62.5%). Peripheral intradermal administration of VEGF-C enhanced lymph node fragment regeneration (70.8%) better than injections of tetanus toxoid (41.6%) or *Streptococcus suis* (62.5%). Lymph node fragment regeneration also occurred in an irradiated porcine model of lymphadenectomy under VEGF-C administration (66.6%).

**Conclusions:**

The present findings provide a pre-clinical proof-of-concept for a possible simplification strategy for current operative procedures of autologous lymph node transplantation.

Level of evidence : Not gradable

## Introduction

Lymphoedema is one of the few diseases that has entered this millennium without an established cure. It causes significant physical and psychological morbidity to affected individuals, including increased susceptibility to infections [1]. The burden of lymphoedema, which has multiple causes [2], is particularly dramatic in countries without the necessary resources for multimodal patient management. Approximately 10–30% of patients undergoing oncologic lymph node resection and/or radiation (e.g. for treatment of breast or prostate cancer) develop lymphoedema of the limbs [3]. Symptoms of secondary lymphoedema can occur because of lymph node loss. These secondary lymphoid organs strategically collect lymphatic fluid to adapt our immune response to the fluid samples they receive from their drainage areas. Interestingly, lymph nodes do not only possess the ability of inducing potent immune responses, but also reabsorb lymphatic fluid within their own blood capillary network after filtering it [4]. Together, these processes ensure resorption of approximately 60% of the total lymphatic fluid in healthy individuals [4]. Primarily, palliative lymphoedema management is based upon physiotherapy and compression techniques. Other palliative concepts comprise radical fatty tissue liposuction associated with lifelong post-operative compression [5]. Even though additional surgical concepts targeting lymphatic disfunction do exist, evidence of their efficacy is still limited [6]. Research on preventive supermicrosurgical anastomosis of lymphatic vessels and venules is currently limited to small clinical studies without control groups [7, 8]. One of the greatest issues in lymphatic-vessel surgery is the correct targeting and exact positioning of suitable vessels prior to surgical anastomosis. Technical efforts have been made allowing an accurate pre-operative mapping of the patients’ lymphatic [9] and venous anatomy [10], with little or no invasiveness and very promising results. Other surgical concepts are based on the autologous transplantation of vascularized free flaps containing lymph nodes. This surgical approach is currently used for patients with secondary lymphoedema e.g. after oncologic lymphadenectomy and/or radiation [11]. Preliminary studies propose the regional use of these same flaps for lymphoedema prevention e.g. in the groin [12]. From a physiological point of view, this approach aims to substitute resected or destroyed nodes potentially restoring function. Although concerns have been raised over donor site morbidity [13], some clinical studies have reported good results [14]. Nevertheless, little basic research has been done on this subject. This is possibly attributable to the lack of understanding of postsurgical lymphatic regeneration processes as well as limitations in available animal models [15]. Whilst rodent models are common, swine animal models present a much wider functional overlap with human immunology and anatomy [16, 17]. An important aspect in surgical research is the striking structural similarity between porcine and human skin/subcutaneous fatty tissue [18]. Current large animal models usually do not reproduce impaired subcutaneous tissue regeneration e.g. of patients with secondary lymphoedema who have previously been subjected not only to surgical but also to radiation treatments.

The aim of this series of pilot studies was to provide a proof of concept for autologous lymph node transplantation techniques without using microsurgery. Additionally, we aimed at evaluating whether fragmenting harvested lymph nodes before transplantation possibly increased the transplanted areas whilst reducing donor site morbidity. Therefore, we divided the study into three phases.

1. First, we compared several methods of lymph node fragmentation. All tested methods were previously published. These included sagittal (butterfly) [19], sliced (tandem-salami) [20, 21], and transversal fragmentation with [22, 23] and without [19] preservation of the lymph node capsule.
2. Later, we compared the effects of the peripheral administration of several immune stimulants (vaccines, bacteria, or growth factors) on lymph node fragment regeneration.
3. Finally, we aimed at refining a different porcine animal model [23] to mimic tissue scaring as in patients subjected not only to oncologic lymphadenectomy but also to additional radiation therapy. We sought to understand if avascular regeneration was still possible in these adverse conditions.

## Material and methods

The present study comprised three consecutive phases as shown in Table 1. In the first phase, a swine model for bilateral *lymphadenectomy* (minipig model *Lx*) without additional radiation was used to comparatively assess previously published techniques of avascular lymph node fragmentation (sagittal-butterfly [19], sliced tandems-salami [20, 21], transversal fragmentation with capsule [22, 23], and transversal fragmentation after capsulectomy [19]). In the second phase, the effects of several immune stimulants on lymph node fragment vitality rates were comparatively assessed. The used immune stimulants were a vaccine, a bacterium, and a growth factor called vascular endothelial growth factor-C (VEGF-C), a ligand for the endothelial cell-specific tyrosine kinase receptors VEGFR-2, and VEGFR-3 [24]. The third phase of the study included an additional minipig model for both *lymphadenectomy and radiation* (minipig model *LxRx* [23]) and evaluated the occurrence of fragment regeneration after stimulation with VEGF-C.

**Table 1:**
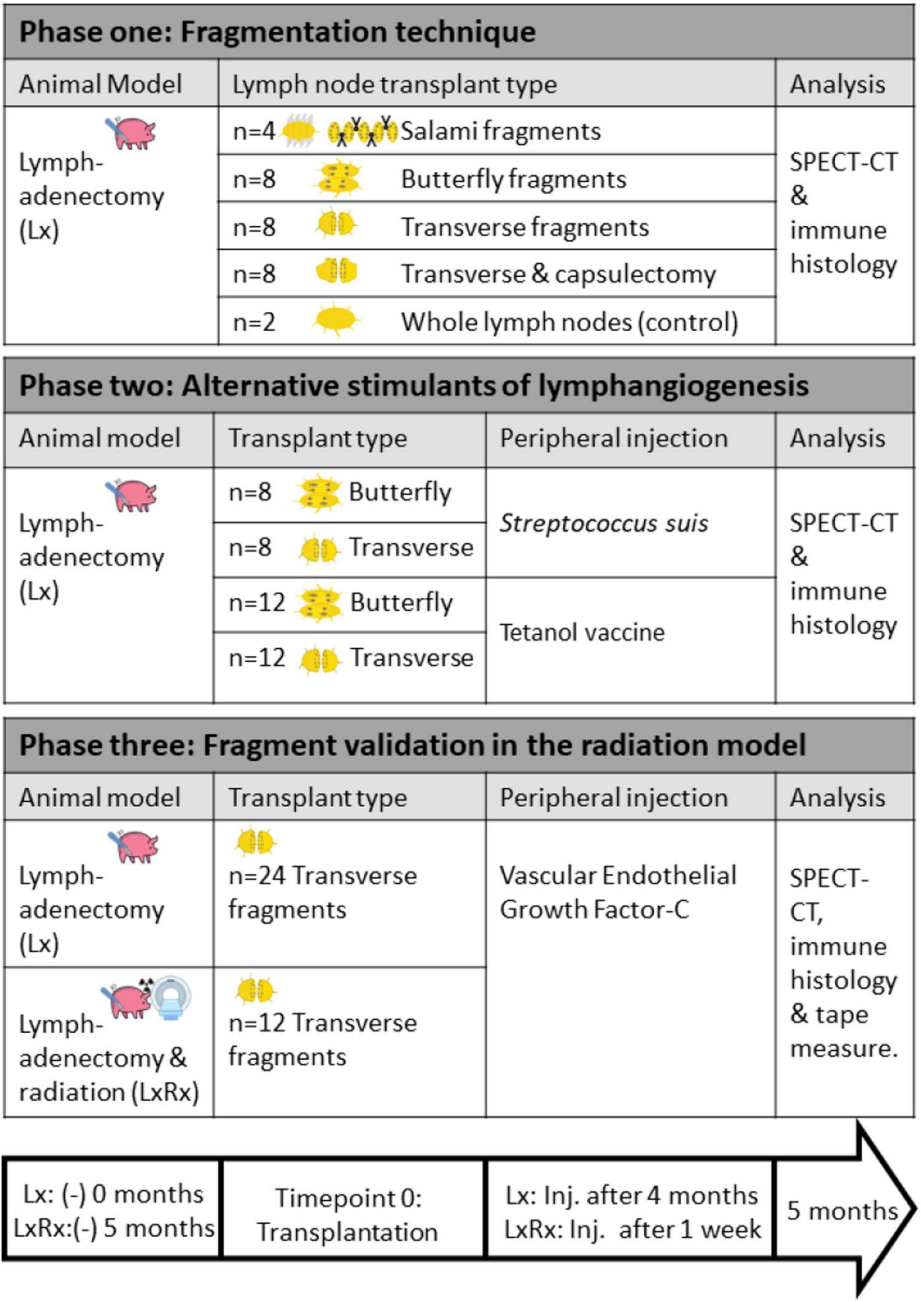
Overview of the study including a timeline (lower arrow)

All operations included in this study were carried out on 33 Göttinger minipigs (29 females and 4 males; 8–12 weeks of age) over five consecutive years by a single surgeon (C.H.). Animals weighted 6 to 12 kg and were obtained from the special pathogen-free facility of the University of Göttingen (Relliehausen, Germany). Housing took place in our facilities under specific pathogen-free (SPF) conditions. Pigs were fed mixed pig food and water ad libitum. Experiments were only started after an adaptive phase of 2 weeks. Premedication and anesthesia were performed as previously described [22, 23, 25]. Post-operative wound care consisted of wound disinfection with aluminium spray (Pharmamedico GmbH, Machern, Germany) applied twice a day for 2 weeks. The animal facilities and all experimental procedures were in accordance with the National Institutes of Health and Association for the Assessment and Accreditation of Laboratory Animal Care guidelines and were approved by the Institutional Animal Care and Use Committee (Ref. Nos. 509.6–42,502-04/809 and 509.6–42,502-07/1402), the Lower Saxony research ethics committee.

To create the minipig model *Lx*, preoperative injection of Berlin Blue dye (LCA GmbH, Apolda, Germany) was given intradermally above the hind hooves for lymphatic visualization. Thereafter, the single superficial inguinal lymph node was resected bilaterally. The fatty tissue of the groin was excised in an oval of 10 cm width and 3 cm height from the abdominal fascia to the piglet’s skin. About 3-mm subcutaneous tissue was left intact for the prevention of skin necrosis. Nine animals were randomly assigned to the above-mentioned lymph node fragmentation techniques (see also Table 1). When performed, capsule resection was made sharply with fine instruments to prevent tissue concussion. Transplants were sutured with non-resorbable colored stitches to facilitate their identification after finishing the experiments. The final timepoint included a combined single-photon emission computed tomography and transmission computed tomography (SPECT-CT) visualization of the lymphatic reconnection of the fragments to the periphery and a histological analysis of the fragments (hematoxylin–eosin) in analogy to a previous publication [22]. As later described, special attention was given to a conserved nodal microarchitecture and presence of Technetium 99 m (Tc-99 m)-nanocolloid/Berlin Blue within the sinuses, as signs of reconnection and fragment vitality.

A preliminary evaluation of results was performed at the end of the first phase, according to SPECT-CT-lymphoscintigraphy (SPECT-CT-LS) and histological assessment of the regeneration rates of the different fragmentation methods. Only the two best fragmentation techniques leading to the highest rates of transplant survival were further used in the second phase of the study.

In this phase, an autologous transplantation of butterfly fragments in the right groin and transverse fragments with capsule in the left groin was performed in model *Lx* (four animals with a total of *n* = 8 fragments of each type). Four months following transplantation, a single intradermal injection of 1 mg/ml suspension of *Streptococcus suis* (Tierärztliche Hochschule, Hannover, Germany) was administered proximal to both hind hooves. One month after this antigen stimulation, animals were sacrificed, and SPECT-CT-LS and histology analyses were performed.

Another group of six minipigs (*Lx* model) was analyzed in this second phase with a different immune stimulant. Four months after bilateral transplantation of butterfly (*n* = 12 fragments) or transverse fragments with capsule (*n* = 12 fragments), bilateral injections of 40 IE tetanus toxoid in 0.5 ml NaCl (Tetanol, Novartis Behring, Marburg, Germany) were given intradermally immediately proximal to each hind hoof. The animals were sacrificed 1 month after this vaccination and SPECT-CT-LS and histological analyses were performed.

To compare the effects of the above-mentioned stimulants with the effects of growth factors, a transplantation of transverse fragments with capsule was conducted bilaterally in the model *Lx* (six animals with a total of *n* = 24 fragments). Four months after transplantation, injections of 10 μg VEGF-C (ReliaTech, Wolfenbüttel, Germany; 5 ml NaCl 0.9%) were administered in the medial aspect right above both hind hooves. The animals received four bilateral intradermal injections in a week (total of 80 μg VEGF-C/animal) and were sacrificed 1 month later.

In the third phase of this study, a different and more complex animal model was used. Our unpublished observations revealed a high rate of wound complications when pigs underwent radiation before surgery. Therefore, minipig models *LxRx* (eight animals) were first subjected to resection of the groin lymphatics as described for minipig model *Lx*. Additionally, the popliteal nodes and the entire fatty tissue were resected without traumatizing the popliteal blood vessels. Wounds were left to heal over 10 days before the radiation of the right groin was performed in sedated animals with a single dose of 15 Gy [23]. A Primus linear accelerator (Siemens Healthcare, Erlangen, Germany) was used to perform radiotherapy with 6-MV photons in a supine position. The irradiated area comprised the region between the midline and the outer aspect of the right groin, and from the gluteal area to the line passing through both caudal mammillae. A silicone gel pad was placed as a “bolus” on the skin over the right groin to compensate for the skin-sparing property of the photons and to include dermal lymphatics [26]. The left groin (future donor site) was protected with a plumb shield.

With a minimal injury to surrounding tissues, the left groin lymph node was taken after 5 months. The two transverse fragments were fixed contralaterally with non-resorbable sutures and perioperative care occurred as described previously [25]. In the week following transplantation, four intradermal injections of 20 μg VEGF-C (ReliaTech) were given unilaterally above the right hind hoof (total of 80 μg VEGF-C/animal). Five months after transplantation, minipig models *LxRx* were sacrificed and analyzed with SPECT-CT-LS. This method consists of a combined evaluation of Tc-99 m-nanocolloid single-photon computed tomography and transmission computed tomography. SPECT and CT images were fused, allowing an exact anatomical characterization of the regenerated fragments, their superficial position in the transplanted areas, and their number. This was a qualitative analysis post-mortem. Due to hygienic reasons related to patient care, SPECT-CT images could not be performed in living pigs. After a combined administration of Technetium-99 m-radiotracer and Berlin Blue dye intradermally immediately above the hind hooves, pigs were kept alive under anesthesia for 1 h, then euthanized and transported in sealed plastic bags for SPECT-CT measurements. Thereafter, histologic probes were traced percutaneously with a hand probe (C-Track automatic equipped with Omni Probe-Gamma, Tc-99 m-collimation, AEA Technology QSA, Brunswick, Germany), and the transplanted areas were taken for histologic analysis, even when no signal/Berlin Blue dye was observable. An additional assessment of tape volumetry measurements was performed as later described.

All tissue samples were cryopreserved [22] and a time interval of a few days was planned to allow reduction of radioactivity. Visualization of the intranodal architecture and distribution of lymphocytes as well as the presence of subcapsular sinuses was considered typical for regenerated and reconnected lymph node fragments. Lymphatic sinuses were also analyzed for the presence of blue pigmentation as a sign of exposure to peripherally injected Berlin Blue. In heterogenic findings with partial intracapsular fibrosis and fatty degeneration within vital lymph node fragments, lymph nodes were only considered necrotic if degenerative features prevailed and no blue dye caption was observable.

Tape volumetry was only performed in model *LxRx*. Immediately prior to lymphadenectomy, hind limb volumes of anesthetized animals were measured with a flexible tape at 4-cm intervals starting at the tip of the external claw. After 5 months, another measurement was conducted intraoperatively prior to fragment transplantation. A third measurement took place 10 months after lymphadenectomy and radiation (5 months after transplantation) shortly before sacrificing the animals. The left limb was used as control. Measurements were expressed as increased volume percentage in comparison to the control limb at a definite timepoint (intraindividual comparison). As young piglets grew in size between measurements, a direct comparison between all timepoints was not possible. Percentages of relative volumes were calculated according to the following formula:

$$\mathrm{Vol }(\mathrm{\%})={A}_{\mathrm{ratio}}\times 100$$

The significance of this volumetric data progression was analyzed using chi-square tests. A value of *p* < 0.05 was considered significant. Graphic analysis of these volume changes was performed using SPSS version 22.0 (SPSS Inc., Chicago, IL, USA) and GraphPad Prism version 8.4.0 (GraphPad Software Inc., San Diego, CA, USA).

## Results

**Fig. 1:**
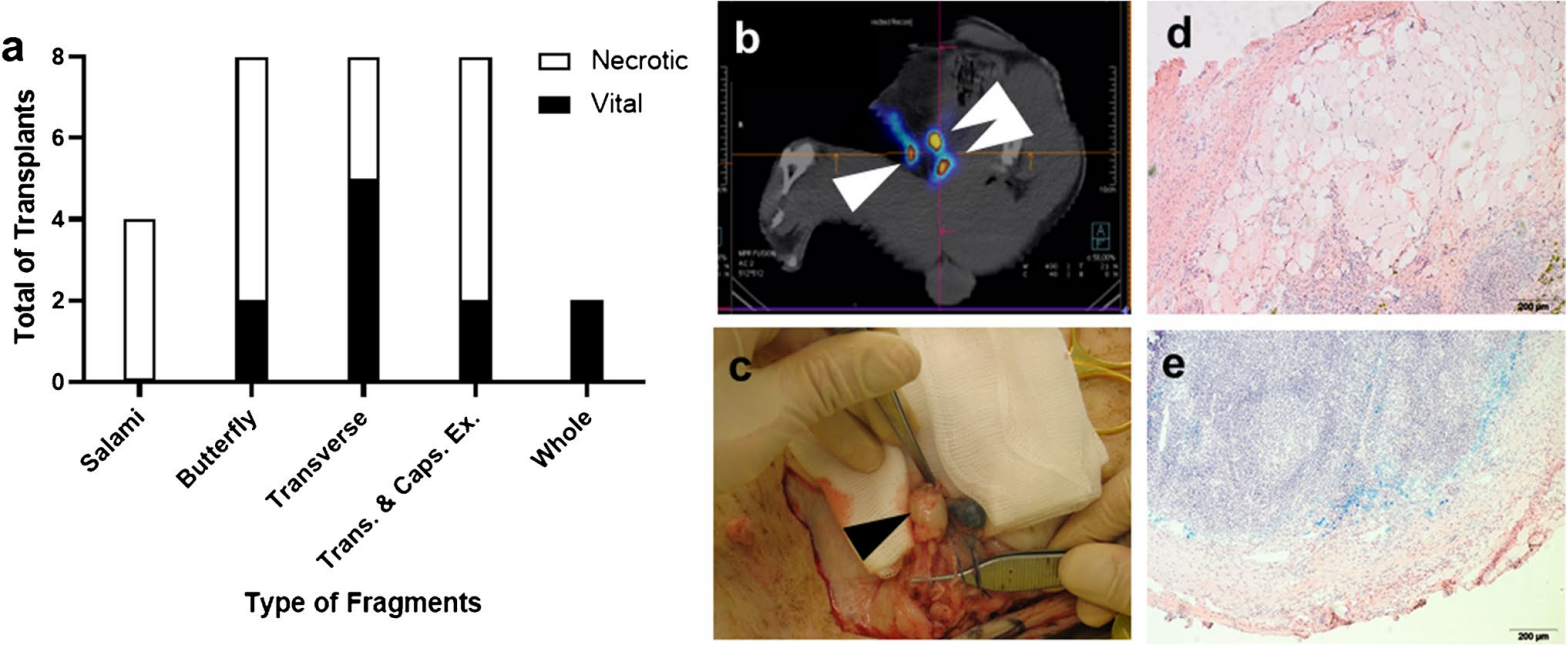
Lymph node fragmentation type affects transplant survival and lymphatic regeneration in the minipig: **a** Outcome of lymph node fragment regeneration in absolute numbers according to different fragmentation procedures. Each fragmentation type was tested bilaterally in two pigs except for the whole lymph node control tested bilaterally in a single animal. The salami fragmentation technique generated one tandem per resected lymph node (*n* = 4); the butterfly and transverse cuts created two fragments per donor node (*n* = 8 each). **b** SPECT-CT imaging (axial plane) of three regenerated lymph node fragments—one in the right groin and two in the left—following transverse cuts maintaining the capsule (white arrowheads). **c** Macroscopic aspect of the same specimen on the right side. Notice the necrotic fragment on the right proximal aspect of the groin lacking the draining function from the periphery and without caption of Berlin Blue (black arrowhead). **d** H&E staining of a cross-section with a necrotic fragment of salami type featuring fatty degeneration. **e** H&E staining of a cross-section with a vital fragment of transverse type featuring blue-stained sinuses and germinal centers. Bar: 200 µm

In the first phase of the study, the minipig model of bilateral lymphadenectomy was created with the objective of better understanding the mechanisms of lymph node fragment regeneration and comparing several fragmentation methods (Fig. 1a). Subsequent analysis demonstrated that transverse cuts without resection of the lymph node capsule achieved the highest level of regeneration (*62.5%* regeneration; 5 vital/*n* = 8 transplants), but still lower than avascular transplantation of whole lymph node as controls (*100%* regeneration; 2 vital/*n* = 2 full nodes). Butterfly cuts had regeneration rates comparable to transverse fragments after complete resection of the capsule (*25%* regeneration; 2 vital/*n* = 8 transplants respectively). SPECT-CT imaging of the animals after 5 months demonstrated how vital fragments were connected to the distal lymphatic system. We were able to establish the exact location of the nodes due to overlapping imaging of lymphoscintigraphy (LS) and computed tomography in SPECT-CT (Fig. 1b). Macroscopic analysis of the Berlin Blue–stained fragments was congruent with SPECT-CT imaging results (Fig. 1c). Transplanted fragments were fixed with non-resorbable sutures. Therefore, all tissue areas within suture marks were analyzed histologically, independently of whether they were blue or not, or positive/negative for Tc-99 m. Transplants lacking a positive signal on the gamma probe showed histological necrosis and sometimes partial tissue resorption (Fig. 1d). All samples with positive gamma probe signal and corresponding to hot spots on the SPECT-CT-images showed organized lymph node tissue in histological analysis (Fig. 1e). Occasionally, blue staining was only present after cutting the fragments, but not evident on the surface of the transplanted organs. Thus, the gamma probe provided a more reliable method of detecting vital fragments compared to sole macroscopic examination. Porcine lymph nodes are inverted; therefore, unlike human nodes, they receive the afferent lymph through the hilus and send the filtered efferent lymph peripherally around the capsule [16]. This ensures a much more efficient distribution of antigens with dendritic cell exposure throughout all lymph node compartments and probably explains why these animals are able to have prolonged contact with pathogens, e.g., in the mud, without permanently risking illness. This peculiar anatomy also causes peripherally injected blue dye to flow first to the centre of the node and later to the surface. When cutting the transplants to a 1 cm size for cryopreservation of the samples until technetium radioactivity had vanished, internal blue dye could also be seen macroscopically when present. Therefore, no discrepancy was observed between SPECT-CT-LS analysis and macroscopic examination, even when the lymph node fragment surface was not clearly blue. Lymph nodes with blue staining in their inner part without macroscopic evidence of blue dye on their surface were considered viable and classified as such.

**Fig. 2:**
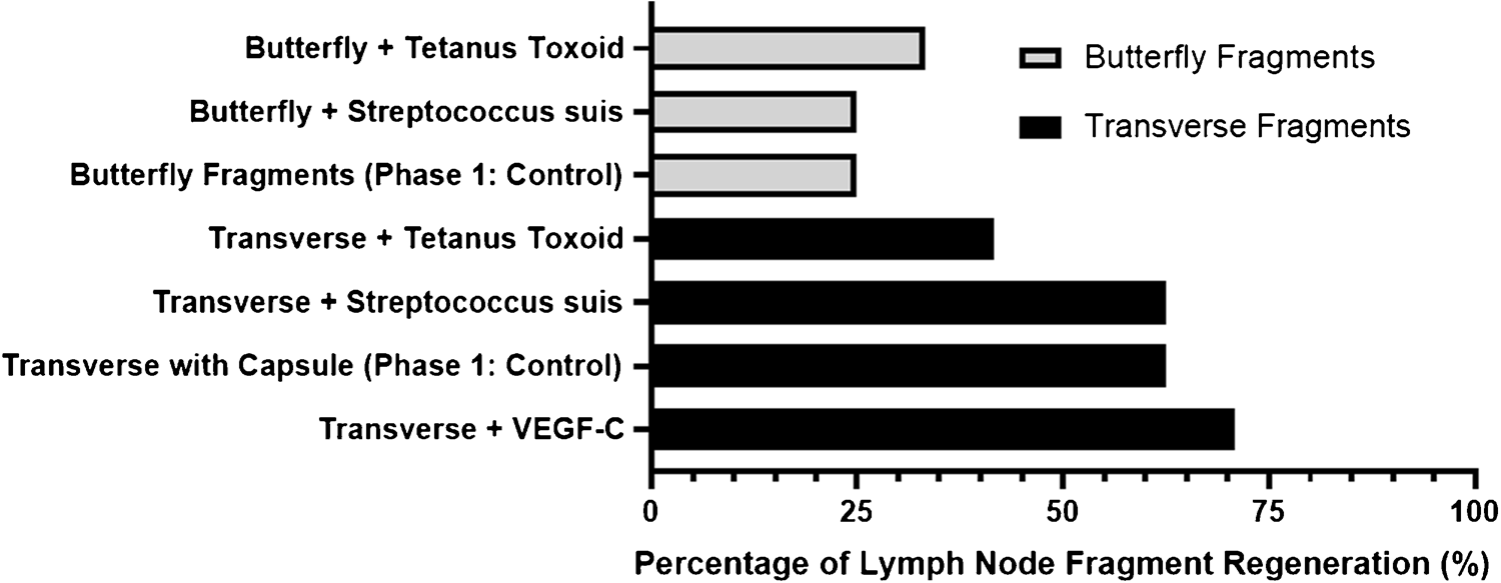
Peripheral stimulation of transplanted fragments with VEGF-C induces higher regeneration rates than stimulation with tetanus toxoid or *Streptococcus suis*. Regeneration rates in percent of butterfly (grey columns) and transverse fragments (black columns) after stimulation with tetanus toxoid or *Streptococcus suis* compared to regeneration rates without additional stimulants (controls) and of transverse fragments after four postoperative VEGF-C injections

In the second phase of the experiments, we investigated alternative processes that could potentially stimulate lymph node fragment regeneration. We sought to compare the effects of postoperative injections of tetanus toxoid (Tetanol vaccine) and *Streptococcus suis* (antigen) injections on both butterfly and transverse lymph node fragmentation techniques (model *Lx*). In these experiments, no significant advantage could be seen in the number of regenerated fragments after vaccination with Tetanol or stimulation with *Streptococcus suis* (Fig. 2). Nevertheless, the advantages of sectioning the nodes transversely (with preservation of the capsule) compared to the butterfly cuts were confirmed. Therefore, subsequent stimulation with VEGF-C injections (third phase of the study) was performed exclusively with the transverse fragmentation technique preserving the capsule (Fig. 2). VEGF-C stimulation seemed to improve the regeneration of transverse fragments (*70.8%* regeneration; 17 vital/*n* = 24 fragments) compared to transverse fragment transplantation without additional stimulants (*62.5%* regeneration; 5 vital/*n* = 8 fragments) (pilot study phase 1), or immune stimulation with *Streptococcus suis* (*62.5%* regeneration; 5 vital/*n* = 8 fragments), or with Tetanol (*41.6%* regeneration; 5 vital/*n* = 12 fragments) (pilot study phase 2).

**Fig. 3:**
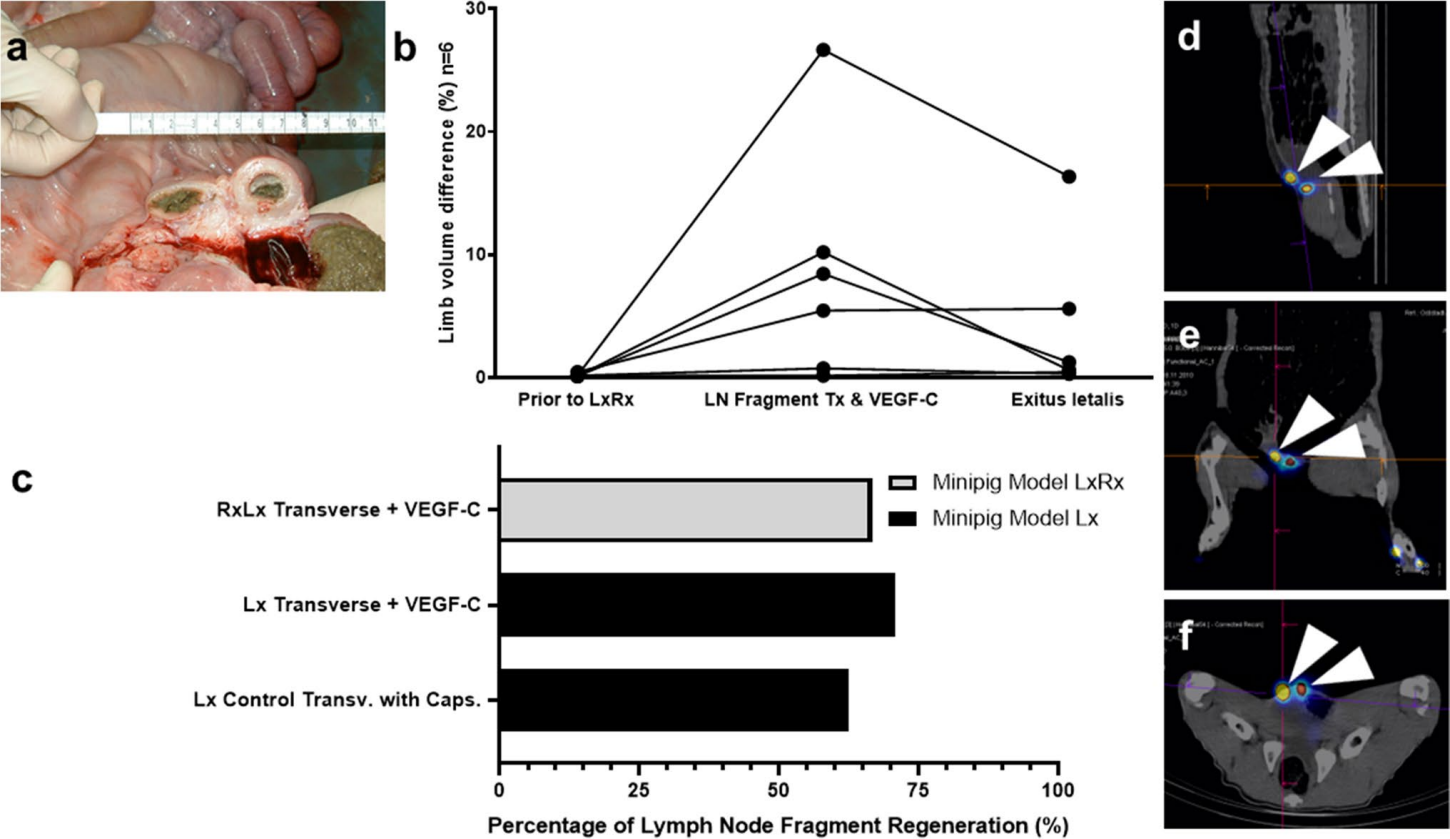
Minipig model following lymphadenectomy and radiation. **a** Autopsy showing ileus and luminal stenosis of the caecum following radiation of the right groin. **b** Volumetric differences between the right (therapy) and left (control) hind limbs. The first timepoint was prior to the model of lymphadenectomy and radiation (*LxRx*). The second measurement occurred at 5 months upon autologous transplant of lymph node fragments. The last timepoint was after 10 months when the animals were sacrificed. **c** Regeneration rates in percent of lymph node fragments after short-term VEGF-C stimulation in the operated and irradiated (*LxRx*) swine model (grey), and in the lymphadenectomy (*Lx*) model (black) with or without VEGF-C stimulation. **d** Sagittal, **e** coronal, and **f** axial planes of a SPECT-CT imaging of the two regenerated fragments in the groin (white arrowheads) in the *LxRx* model

The observed regeneration of avascular transverse lymph node fragments in the *Lx* pig model left the question of a possible regeneration in previously irradiated tissue open. To analyze this, a new minipig model *LxRx* was developed to mimic the situation of oncologic patients subject to lymph node resection and radiation. We noted the development of complications following irradiation of the pre-operated sites. These included the occurrence of spontaneously resolving wound seromas in the first week after radiation (2 weeks after lymphadenectomy) in two animals of eight. Additionally, we observed later complications of radiation as recurrent ileus symptoms in a male animal after 1 month (Fig. 3a). Terminal-urethral fibrosis with chronic urine retention was discovered in another animal after 2 months. In accordance with the German legal specifications for animal protection, both animals were euthanized, leaving this experimental group with a total of six animals.

Macroscopic limb swelling, although clearly present after radiation, resolved with time in most cases (Fig. 3b). Therefore, later transverse lymph node fragment transplantation from the left (control site) into the right groin did not cause a statistically significant difference in hind limb volume in this model (Fig. 3b), as only *acute* lymphoedema occurred. Nevertheless, the minipig model *LxRx* consistently reproduced tissue scarring similar to pre-operated and irradiated patients. This tissue finding was objectively present during all lymph node fragment transplantation surgeries 5 months after radiation.

We proceeded to investigate the regeneration of lymph node transplants in the six minipig models *LxRx*. Surprisingly, despite massive tissue scarring, reconnection of lymph node fragments to peripheral lymphatics could still be observed in many cases (*66.6%* regeneration; 8 vital/*n* = 12 transplanted fragments). The previous model *Lx* without radiation was used as a positive control. Regeneration rates in the minipig model *LxRx* were somewhat lower than in the minipig model *Lx* (without radiation) even with both receiving VEGF-C stimulation (Fig. 3c). Nevertheless, regeneration rates seemed to be improved in comparison to transverse fragment transplantation without additional stimulants (pilot phase 1) (*62.5%* regeneration; 5 vital/*n* = 8 transplants) or regeneration rates in minipig model *Lx* using other stimulants or other fragmentation techniques.

Regeneration rates seemed to be superior in animals with chronic macroscopic oedema compared to irradiated animals without macroscopic swelling. The two animals with a percentual increase in a volume of 26.6% and 10.2% prior to transplantation (Fig. 3b) exhibited regeneration rates of 100% of the transplanted fragments (4 vital/*n* = 4 fragments). SPECT-CT imaging and histological sections of regenerated lymph node fragments were comparable to regenerated fragments in the model without prior tissue irradiation (Fig. 3d–f).

### Discussion

More than a dozen different operative interventions have been proposed to date for the management of lymphoedema [27]. Concepts based on lymph node flaps with blood vessel microanastomosis appear to have superior results. However, these operative procedures are technically demanding and apparently pose some risk of donor site morbidity [13]. Additionally, these advanced techniques cannot be performed in most developing countries due to the intraoperative need for a high-resolution microscope [28]. However, the worldwide incidence of the lymphoedema pandemic should motivate researchers to seek operative solutions also applicable in areas of the globe with financial restrictions.

We analyzed this previously reported simplified technique of fragmentation of autologous lymph nodes and transplantation into the subcutaneous tissue [23]. The importance of the “cross-talk” between fatty cells and lymph nodes has been described [29], although it is still poorly understood.

In this study, we demonstrated that the type of fragmentation crucially influences lymph node regeneration processes. This illustrates the central role of lymph node stromal cell survival after avascular transplantation [30]. Initial diffusion processes promote survival of lymph node stroma [30] and induce migration of dendritic cells and B- and T-lymphocytes [31] from the bloodstream, creating the necessary environment for regeneration of the organ and its lymphatic afferents [24] and restoring its immune function [24]. Nevertheless, the previously reported advantages of several published techniques of fragmentation (salami [20, 21], butterfly [19], transverse including capsulectomy [19]) could not be confirmed. In our study, the presence of the capsule seemed to positively influence lymph vessel regeneration as lymph node fragments with capsule regenerated and reconnected better than in the absence of it. In regenerated fragments, internal nodal architecture was present illustrating immune competence. Additionally, previous observations have shown that gradients of lymphatic growth factors in the subcutaneous tissue, including VEGF-C, positively influence lymphatic regeneration [32] and significantly increase the absolute number of vital fragments after lymph node transplantation [26]. Nevertheless, it remained unclear if other forms of immune stimulation also could improve lymph node fragment regeneration.

In the second phase of these pilot studies, we could demonstrate that immune stimulation with tetanus toxoid or *Streptococcus suis* did not appear to exert an influence on fragment regeneration percentages. This seems to demonstrate relative independence between acute immune processes and lymphangiogenic stimulation [33]. Chronic inflammatory diseases and chronic autoimmune conditions seem to increase lymphangiogenesis [33]. Therefore, the long duration of immunologic stimuli might be relevant for lymphangiogenic activation [34] whereas VEGF-C stimulation might have effects within a shorter time period [32]. This might explain why regeneration was improved in the presence of VEGF-C. However, patients with secondary lymphoedema can develop the condition as a result of cancer treatments. Therefore, the clinical use of VEGF-C should be performed with caution and oncologic follow-up care should be intensified in order to prevent cancer relapse through potential reactivation of the vascular supply of dormant malignant cells [35].

In the third phase of the study, the proposed porcine animal model *LxRx* of surgical fibrosis and tissue irradiation encountered some difficulties due to swine sensitivity to radiation. Therefore, we advise the use of female specimens for this big animal model to prevent exposure of the penile urethra to conventional photon irradiation. Also, limb volume had to be expressed as a ratio between the operated and the control limb due to the growth in the size of the young animals during the study. Surprisingly, it did not substantially differ on both sides despite the aggressiveness of the procedure. This might have occurred due to the relative shortness of the limbs in minipigs in proportion to their bodies [15]. Big animal models in sheep and horses have been described that might be less efficient in regenerating lymphatic function and better for the creation of chronic lymphoedema due to proportionally long limb size in these species [36]. Functional competence of fragments and successful induced lymph vessel growth in spite of prior tissue radiation could be illustrated through radionuclide transport in SPECT-CT imaging and was confirmed histologically in our swine model.

## Conclusions

Transplantation of vascularized free flaps containing lymph nodes already constitutes a promising surgery for secondary lymphoedema. In minipig models for lymphadenectomy without/with tissue radiation, we could show that a percentage of transplanted avascular lymph node fragments were able to regenerate and reconnect to the peripheral lymphatic drainage pathways. The effects of tetanus toxoid, *Streptococcus suis*, or the growth factor VEGF-C could be documented e.g. by SPECT-CT imaging. The simplified transplantation procedure with transverse-cut fragments seems to induce lymphangiogenesis in the majority of cases, is technically accessible, and could potentially reduce donor site morbidity. Occurrence of lymph node fragment regeneration and functional reconnection in the minipig models could suggest potential therapeutic benefits of this surgical concept in the management of human lymphatic diseases in the context of oncologic lymphadenectomy and/or radiation.

## Data Availability

The data supporting the findings of this study is available from the corresponding author, upon reasonable request.
